# Atypical parkinsonism and intrathecal anti-glutamic acid decarboxylase antibodies – an unusual association: a case report

**DOI:** 10.1186/s13256-020-02412-x

**Published:** 2020-06-30

**Authors:** Giovanni Stefanoni, Anna Formenti, Lucio Tremolizzo, Andrea Stabile, Ildebrando Appollonio, Carlo Ferrarese

**Affiliations:** Neurology Unit, “San Gerardo” Hospital and School of Medicine and Surgery, University of Milano – Bicocca, Milan-Center for Neuroscience (NeuroMI), Monza, Italy

**Keywords:** Atypical parkinsonism, Anti-GAD antibodies, Intrathecal antibodies

## Abstract

**Background:**

Immunological causes of parkinsonism are very rare and usually characterized by early presentation, poor response to levodopa, and additional clinical features.

**Case presentation:**

We describe a 58-year-old white man who presented with a 1-year history of gait disturbance with disequilibrium leading to falls. We report an association between parkinsonism and presence of anti-glutamic acid decarboxylase antibodies in his cerebrospinal fluid, discussing clinical presentation and follow-up.

**Conclusions:**

Besides the possibility of a casual association, this case allows us to hypothesize an alternative pathophysiological mechanism of parkinsonism implying interference with glutamic acid decarboxylase and gamma-aminobutyric acid functions, eventually resulting in basal ganglia circuit dysregulation.

## Introduction

Immunological causes of parkinsonism are very rare and usually characterized by early presentation, poor response to levodopa, and additional clinical features, such as dementia, postural instability, eye movement abnormalities, and cognitive impairment. A series of patients was described as having parkinsonism plus syndromes associated with anti-LG1 or other anti-neuronal antibodies directed against uncharacterized antigens [1]. Here we report an association between parkinsonism and the presence of anti-glutamic acid decarboxylase (GAD) antibodies in the cerebrospinal fluid (CSF).

## Case presentation

We describe a 58-year-old white man who presented with a 1-year history of gait disturbance with disequilibrium leading to falls. His medical history was significant only for past alcohol abuse, interrupted approximately 5 years before. His family history was negative per movement disorder and, in general, for neurodegenerative conditions. A neurologic examination showed mild hypomimia, mild hypophonia, mild dysarthria, saccadic pursuit eye movements, asymmetric mild-moderate bradykinesia with right prevalence, moderate muscle rigidity of upper limbs, moderate-severe rigidity of lower limbs, and shuffling gait. Formal neurophthalmological evaluation failed to highlight further signs, in particular, the “round the houses” sign was absent [2] as well as supranuclear vertical gaze palsy. He could walk without support but pull test was positive. His Unified Parkinson’s Disease Rating Scale-III (UPDRS-III) score was 44/104. His Hoehn and Yahr stage was 3. Limb strength, sensitivity, and coordination were normal. No symptoms and signs of dysautonomia were found. His cognitive functions were studied through a complete battery of neuropsychological tests, which highlighted mild deficits in visuo-constructive and executive functions. Brain magnetic resonance imaging (MRI) showed diffuse cerebral atrophy involving both supratentorial and subtentorial regions, with more pronounced involvement of bilateral parieto-frontotemporal lobes, cerebellar worm, and midbrain; no alterations were shown in the basal ganglia or cerebellar deep nuclei; furthermore, no evidence of cerebrovascular disease was noted. A spinal cord MRI was normal. Electroencephalography showed mild diffuse slowing of electric cortical activity without periodic waves or epileptic discharges. Electromyography documented mild polyneuropathy. ^123^Iodine fluoropropyl-CIT single-photon emission computed tomography (FP-CIT SPECT) showed normal dopamine transporter (DAT) uptake. ^18^F-fluorodeoxyglucose (18F-FDG) brain positron emission tomography (PET) demonstrated bilateral parieto-temporal hypometabolism. A diagnosis of atypical parkinsonism was made and levodopa therapy was introduced at a dosage up to 400 mg a day, with mild improvement of limb rigidity and bradykinesia but no amelioration of gait stability (UPDRS-III 35/104, Hoehn and Yahr 3). Further increase of levodopa was not possible since he refused, at that time, due to side effects.

A panel of blood examinations was performed in order to search for metabolic or dysimmune causes of parkinsonism. Serum antinuclear antibodies (ANA), extractable nuclear antigens (ENA), anti-neutrophil cytoplasmic antibodies (ANCA), anti-GAD, anti-tissue transglutaminase antibodies, anti-human T-lymphotropic virus (HTLV) 1–2 antibodies, and anti-onconeural antibodies: anti-Hu, Ri, Yo, Ma, amphiphysin, CV2, and paraneoplastic antigen Ma2 (PNMA2) were negative. Genetic testing for spinocerebellar ataxia (SCA) types 1, 2, 3, 6, and 7 was negative as well. Plasma levels of ceruloplasmin, copper, and neoplastic markers were normal, as well as liver function. A lumbar puncture was performed. CSF had normal levels of glucose, proteins, and leukocytes. Oligoclonal bands and onconeural antibodies were negative. Finally, a search for anti-GAD antibodies in CSF by radioimmunoassay gave a positive, albeit low titer, result (2.0 U/ml, normal values under 1.0 U/ml).

At the last follow-up visit, performed 15 months after hospital admission and after completion of an intensive cycle of rehabilitative treatment, he showed mild improvement of gait stability, with no change in limb bradykinesia and rigidity. UPDRS-III was 31/104 and Hoehn and Yahr stage was 2.5. Given the acceptable functional status, the absence of T2 hyperintense or contrast-enhanced brain lesions on MRI, the lack of inflammatory changes in CSF, and the low titer of CSF anti-GAD, we have decided not to introduce immunosuppressive therapy at the moment. Follow-up is currently ongoing for detecting any possible clinical modification in order to decide whether to increase levodopa dosage or opt for potential immunotherapies. Furthermore, we are monitoring our patient for the possibility of the appearance of malignancy.

## Discussion

Anti-GAD antibodies are known to be strongly associated with type 1 diabetes mellitus. Furthermore, a wide spectrum of neurological syndromes has been described in patients with very high levels of serum anti-GAD [3]. Among these syndromes, the most frequent are stiff person syndrome, where serum anti-GAD are present in up to 80% of cases, and cerebellar ataxia. Epilepsy, limbic encephalitis, and other syndromes are less common. Reports of associations between anti-GAD and parkinsonism are lacking. Only a series of the Mayo Clinic described extrapyramidal signs in 16% of patients with anti-GAD antibodies. However, these patients had mild parkinsonism, usually associated with more prominent cerebellar signs or features of stiff person syndrome. Moreover, these patients had anti-GAD positivity in serum [4]. Recently, Sunwoo and colleagues described patients with anti-GAD antibodies in CSF but not in paired serum samples [5]. In this series, the most frequent clinical syndromes were limbic encephalitis and temporal lobe epilepsy, whereas cerebellar ataxia was less frequent and stiff person syndrome was very rare. None of these patients manifested extrapyramidal signs [5]. Polyclonality of anti-GAD antibody may theoretically explain such clinical variability [6], and clinical presentation and anti-GAD titers do not always correlate. Further research in this matter, correlating clinical phenotype to the specific antibody profile, is critical [6].

## Conclusions

To the best of our knowledge, our case could represent one of the few cases of parkinsonism associated with anti-GAD antibodies. Even if we cannot exclude an indirect association, in particular considering the relatively low titer, the possible existence of a pathogenic correlation is intriguing. Early onset, mild response to levodopa, and presence of atypical manifestations, such as oculomotor abnormalities, cognitive impairment, and postural instability are features observed in this case and described in previous reports of autoimmune parkinsonism [1]. The negative result of FP-CIT SPECT excludes damage of dopaminergic nigro-striatal projection. However, compatibly with the reported findings, we cannot completely exclude that the clinical evolution could disclose in the future a picture of either multiple system atrophy or progressive supranuclear palsy.

In any case, these data open the possibility to hypothesize an alternative pathophysiological mechanism of parkinsonism implying interference with GAD function and subsequent gamma-aminobutyric acid (GABA) depletion, with subsequent dysregulation of basal ganglia circuits.

## Data Availability

Data sharing not applicable to this article as no datasets were generated or analyzed during the current study.

## Abbreviations

ANA: Antinuclear antibodies
ANCA: Anti-neutrophil cytoplasmic antibodies
CSF: Cerebrospinal fluid
DAT: Dopamine transporter
ENA: Extractable nuclear antigens
FP-CIT SPECT: ^123^Iodine fluoropropyl-CIT single-photon emission computed tomography
GABA: Gamma-aminobutyric acid
GAD: Glutamic acid decarboxylase
HTLV: Human T-lymphotropic virus
MRI: Magnetic resonance imaging
PNMA2: Paraneoplastic antigen Ma2
SCA: Spinocerebellar ataxia
UPDRS-III: Unified Parkinson’s Disease Rating Scale-III
